# The median effective dose (ED50) of *cis-*Atracurium for laryngeal mask airway insertion during general Anaesthesia for patients undergoing urinary surgery

**DOI:** 10.1186/s12871-020-00982-3

**Published:** 2020-03-19

**Authors:** Xiaohua Wang, Ke Huang, Hao Yan, Fei Lan, Dongxu Yao, Yanhong Li, Jixiu Xue, Tianlong Wang

**Affiliations:** 1 Department of Anesthesiology, Xuanwu Hospital, Capital Medical University, Beijing, 100053 China; 2 Institute of Geriatrics, Beijing, 100053 China; 3 National Clinical Research Center for Geriatric Disorders, Beijing, 100053 China; 4 Department of Urinary surgery, Xuanwu Hospital, Capital Medical University, Beijing, 100053 China

**Keywords:** Sequential method, ED50, *cis*-Atracurium, Urinary surgery, Laryngeal mask

## Abstract

**Background:**

In clinical practice, the laryngeal mask airway is an easy-to-use supraglottic airway device. However, the *cis-*atracurium dosage for laryngeal mask insertion has not been standardised. We aimed to determine the optimal dose of *cis-*atracurium using a sequential method for successful laryngeal mask insertion.

**Methods:**

The cohort study protocol is registered at clinicaltrial.gov (NCT-03668262). Twenty-three patients undergoing elective urinary surgery were sequentially administered *cis-*atracurium doses as follows: 150, 100, 70, 50, 30, and 20 μg·kg^− 1^. The main outcome involved the determination of the response to laryngeal mask airway insertion: ≥16 points and < 16 points indicated “satisfactory” and “unsatisfactory” responses, respectively. The median effective dose was estimated using the mean of the seven crossovers from “satisfactory” and “unsatisfactory” responses. The primary outcome involved the determination of the median effective dose (ED50) of *cis-*atracurium for laryngeal mask airway insertion.

**Results:**

The median effective dose of *cis-*atracurium was 26.5 μg·kg^− 1^ (95% CI 23.6–29.8) using the sequential method. Heart rate was decreased in the 50 μg·kg^− 1^ group compared to that in the 30 μg·kg^− 1^ group at timepoints T7, T8, and T10 (*P* = 0.0482, *P* = 0.0460, and *P* = 0.0236, respectively), but no difference was observed in the 20 μg·kg^− 1^ group. Systolic blood pressure was decreased in the 50 μg·kg^− 1^ group compared to that in the 20 μg·kg^− 1^ group at timepoints T2, T3, and T4 (*P* = 0.0159, *P* = 0.0233, and *P* = 0.0428, respectively). The train-of-four value was significantly lower in the 50 μg·kg^− 1^ group than in the 30 μg·kg^− 1^ group at timepoint T3 (*P* = 0.0326).

**Conclusions:**

The ED50 of *cis-*atracurium was 26.5 μg·kg^− 1^ for laryngeal mask airway insertion.

**Trial registration:**

Clinicaltrial.gov Registry, NCT03668262, Registered on 11 September 2018.

## Background

The laryngeal mask airway (LMA) is a supraglottic ventilation device that is more effective than mask airway for difficult airway management and includes the characteristics of mask and endotracheal intubation [1]. LMA insertion has more advantages than endotracheal intubation: it has little influence on the patient’s circulation during insertion, reduces the dosage of analgesics required to maintain anaesthesia during surgery, and is well-tolerated [2]. Therefore, the laryngeal mask is widely used in elective minor surgery, particularly in minor urinary surgery [3]. The use of muscle relaxants is necessary to improve the conditions of laryngeal mask insertion [4]. Without muscle relaxants, the pharyngeal tissues are not relaxed, and appropriate laryngeal mask placement is difficult owing to resistance to mouth opening and biting [5]. Moreover, muscle relaxation is needed to avoid excessive airway reactivity of laryngeal mask insertion (e.g., laryngeal spasm, hypersalivation, coughing), to reduce laryngeal mask insertion-related complications (e.g., postoperative throat pain), and to reduce the incidence of airway complications such as hypoxia, ineffective ventilation, and sternal muscle stiffness that are induced by opioid analgesics [6–8]. Analgesic and narcotic agents are required in high dosages without the use of muscle relaxants, and an overdose of analgesic agents can inhibit the patient’s haemodynamics [9]. Small doses of neuromuscular blocking agents (NMBAs) may improve mandibular relaxation and shorten the time required for laryngeal mask placement, thereby improving the laryngeal mask placement conditions [10].

*Cis*-atracurium is a non-depolarizing NMBA and has been widely used adjunctively during anaesthesia to facilitate endotracheal intubation and provide a longer duration of muscle relaxation [11]. It is spontaneously degraded at physiological pH via Hofmann elimination, which is an organ-independent degradative mechanism that yields laudanosine and plasma esterase-mediated hydrolysis [12]. Little or no risk is associated with the use of *cis*-atracurium in patients with renal diseases; therefore, it is frequently used in general anaesthesia during urinary surgery. Furthermore, *cis*-atracurium has unique advantages and is approximately three times more potent than atracurium as a muscle relaxant [13]. It has less propensity to induce histamine release, which causes subsequent cutaneous flushing, hypotension, and tachycardia complications [14], and it significantly reduces the use of proinflammatory markers during surgery [15]. However, an overdose of *cis*-atracurium may increase the risk of aspiration, airway obstruction, and delayed recovery. The dosages of muscle relaxants used in trachea intubation vary greatly and range from 10 to 200 μg·kg^− 1^ [16–19]. However, a reasonable dosage of *cis*-atracurium under LMA has not been reported.

This study aimed to determine the median effective dose (ED50) of *cis*-atracurium for laryngeal mask insertion in anesthetised adults using Dixon’s up-and-down method [20] and to determine the dose-response curves for laryngeal mask insertion in urinary surgery.

## Methods

### Patient characteristics

This prospective observational study was approved by the university’s institutional review board (IRB no.: CINI-AD-20180808). All individuals participating in the trial provided written informed consent. The trial is also registered at http://ClinicalTrials.gov (registry no.: NCT-03668262; date of registration: September 11, 2018). The methodology in this study followed the international guidelines for observational studies according to the Strengthening the Reporting of Observational Studies in Epidemiology (STROBE) 2010 statement (Supplementary-STROBE checklist). We recruited 23 prospective consecutive patients who were scheduled for elective minor urological surgery under general anaesthesia between 15 September, 2018, and 30 January, 2019, at Xuanwu Hospital (Beijing, China). All patients met the criteria for the American Society of Anesthesiologists (ASA) Physical Status I–III; Body Mass Index (BMI), 18.5–30 kg/m^2^; age, 20–60 years; predicted operation duration < 180 min; and estimated blood loss < 5 ml·kg^− 1^. The exclusion criteria were as follows: neuromuscular diseases; metabolic diseases; preoperative condition complicated with electrolyte disorders, serious hepatic insufficiency (i.e., liver transaminase level > 40 U·L^− 1^), or renal insufficiency (i.e., serum creatinine level > 1.2 mg·dL^− 1^); serious heart and lung disease; predicted difficult airway; use of preoperative medications that interact with non-depolarising NMBAs (e.g., aminoglycosides, polymyxin, steroids, phenytoin sodium, neuroleptics, carbamazepine); history of allergy to NMBAs; and history of alcoholism or drug abuse.

### Anaesthesia protocol

After the patient entered the operating room, lactate Ringer’s solution was infused at a rate of 5 ml·kg^− 1^·h^− 1^. After 3 min of oxygen supplied via the mask, intravenous sufentanil (dose, 0.25 μg·kg^− 1^; injection time, 30 s) and etomidate (dose, 0.2 mg·kg^− 1^; injection time, 30 s) were administered. The bispectral index (BIS) reaching a value below 60 and loss of the eyelash reflex indicated that the patient had lost consciousness, and the train-of-four (TOF) value was calibrated. The calibration of TOF as the baseline was 100. After calibration, *cis*-atracurium was administered (injection time, 5 s). Three minutes after the *cis*-atracurium injection, and once the BIS reached a value of 40–60, an experienced anaesthesiologist placed a flexible LMA (Teleflex Medical, Wayne, PA, USA; Athlone Co., Westmeath, Ireland) of the appropriate type (LMA® Flexible criteria: 30–50 kg for No. 3; 50–70 kg for No. 4; and 70–100 kg for No. 5). The patients’ responses were jointly evaluated by another anaesthesiologist who was blinded to the *cis*-atracurium concentrations when the LMA was inserted. The tidal volume setting was 7 ml·kg^− 1^, and the respiration rate setting was 12 RR·min^− 1^. If laryngeal mask insertion was unsuccessful, anaesthesia was deepened with further increments of *cis*-atracurium, or an inhalation agent, or both, until the laryngeal mask was tolerated; however, the patient was placed in the “unsatisfactory” group in these cases. Propofol combined with remifentanil was used to maintain the BIS at 40–60 throughout the operation. The range of blood pressure and heart rate (HR) fluctuation did not exceed the 20% baseline value. Body temperature was maintained at > 36 °C using a warm air blanket.

### Cis-Atracurium administration and evaluation of the LMA placement conditions

#### Administration of cis-Atracurium and subdivision of groups

**Fig. 1:**
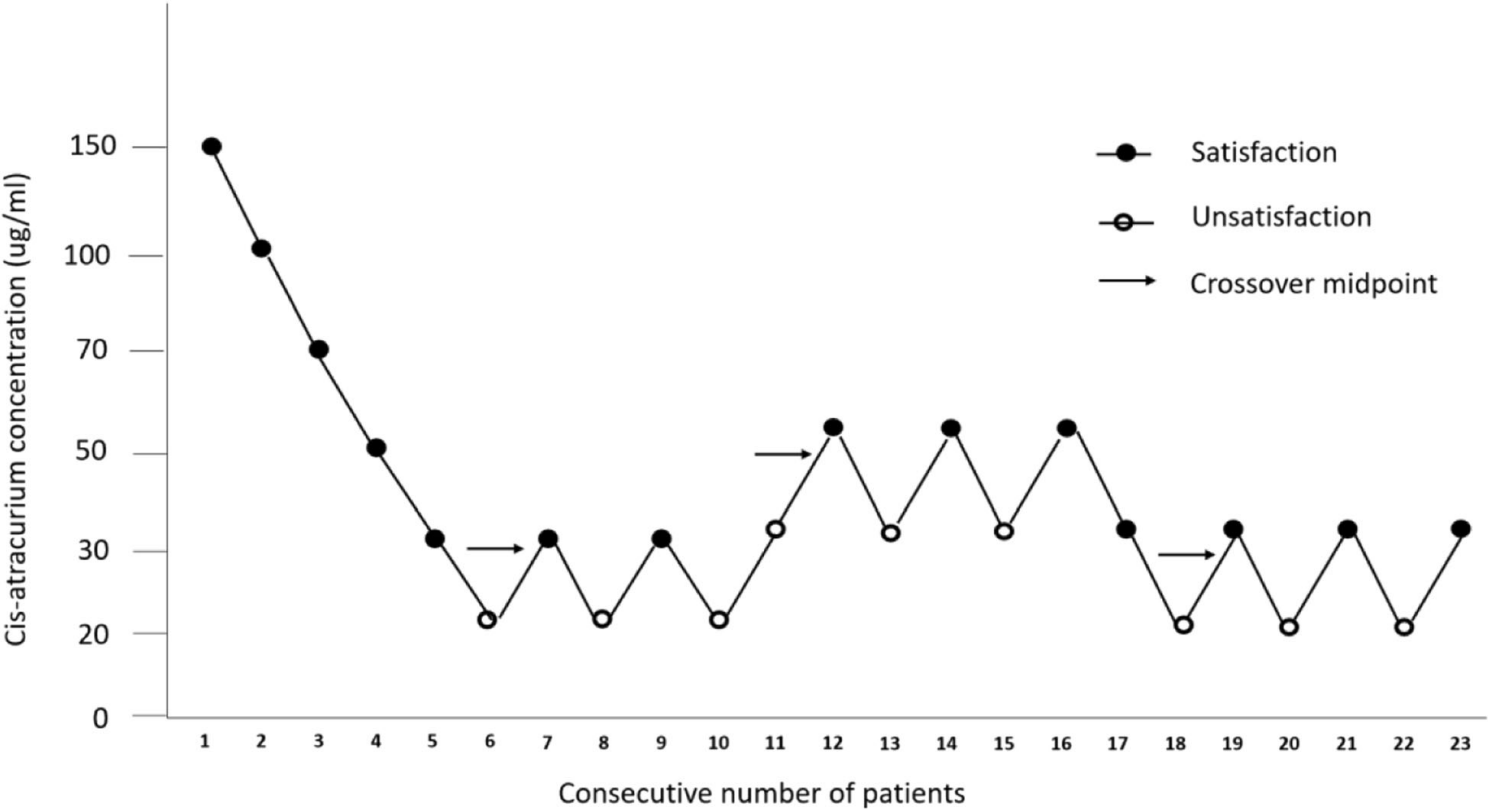
Flow diagram of the patient selection process based on the sequence method

The first patient enrolled in the study was exposed to an initial *cis*-atracurium concentration of 0.15 mg·kg^− 1^. The step size of the concentration was calculated by a common ratio 1.5; thus, the administered doses were 0.1 = [0.15/(1.5)], 0.06667 = [0.15/(1.5)^2^], 0.04444 = [0.15/(1.5)^3^], 20 μg·kg^− 1^ 962 = [0.15/(1.5)^4^], and 0.01975 = [0.15/(1.5)^5^] mg. In the clinical setting, the actual administered doses were 0.15, 0.1, 0.07, 0.05, 0.03, and 0.02 mg kg^− 1^, which are equal to 150, 100, 70, 50, 30, and 20 μg·kg^− 1^ in this study. We categorized the patients into six groups as follows: 150 μg·kg^− 1^ group, 100 μg·kg^−^ 1 group, 70 μg·kg^−^ 1 group, 50 μg·kg^− 1^ group, 30 μg·kg^− 1^ group, and 20 μg·kg^− 1^ group. Depending on the previous patient’s response to the laryngeal mask, using a modified Dixon’s up-and-down method, we adjusted the subsequent dose in the remaining patients [20]. Beginning with the first case of unsatisfactory response, the number of observation units was counted. A “satisfactory-satisfactory” response was noted at seven exchange points. This marked the completion of the test (Fig. 1). When self-contained respiration occurred during the operation, additional doses of *cis*-atracurium were re-administered to the patient.

#### Laryngeal mask placement conditions

The insertion condition was evaluated only when the laryngeal mask was inserted the first time. Using the recognised six-point-three scale proposed by Sivalingam [21], which has been used successfully in previous research, the following were graded: resistance to mouth opening, resistance to insertion, coughing, swallowing, laryngospasm/airway obstruction (including Paw pressure more than 40 mmHg after insertion immediately), and head and body movement. Each item was scored 3 points, 2 points, and 1 point, based on the severity. A full score was 18 points. A score of ≥16 points was considered to be a “satisfactory” response, and a score of < 16 points was considered to be an “unsatisfactory” response (Supplementary) (Table_S1). If the response was “unsatisfactory”, the *cis*-atracurium dosagr was increased in the next patient. After approximately 60 s of successful laryngeal mask insertion, the position and ventilation condition of the laryngeal mask and whether the patient had a “satisfactory” or “unsatisfactory” response were recorded.

### Muscle relaxation (TOF) monitoring, general parameter monitoring, and blood sample analysis

The patient’s upper limb was extended and fixed. After degreasing the skin with alcohol, surface electrodes were placed on the side of the ulnar nerve of the wrist for TOF stimulation monitoring (TOF-Watch; Organon Ireland, Ltd., Swords, Dublin, Ireland). The sensor probe of the TOF monitor was placed between the thumb and forefinger with no resistance between them. When the patient lost consciousness, the calibration scale of T1 was 100, the stimulation current was 45–75 mA, the interval was 5 s, and the frequency was 2 Hz. The nasopharyngeal temperatures, electrocardiography, blood pressure, HR, pulse oxygen saturation (SpO_2_), and BIS of patients were routinely monitored. We set 10 timepoints to record variations during the operation as follows: upon administration of *cis*-atracurium (T1), and 3 min (i.e., at LMA insertion) (T2), 10 min (at the beginning of the operation) (T3), 20 min (T4), 30 min (T5), 40 min (T6), 50 min (T7), 60 min (T8), 70 min (T9), and 80 min after the administration of cis-atracurium (T10). Systolic blood pressure (SBP), diastolic blood pressure (DBP), HR, SpO_2_, BIS, TOF and airway peak pressure (Paw peak pressure) were recorded at each timepoint. Intraoperative haemodynamic changes and adverse reactions such as cough, respiratory depression, dizziness, nausea, and vomiting were also recorded.

### Statistical analysis

The ED50 of *cis*-atracurium was determined by calculating the midpoint concentration of the crossover point from the “satisfactory” or “unsatisfactory” responses. To facilitate the dose-response analysis, the laryngeal mask insertion conditions were recorded as dichotomous outcomes. A probit analysis linear regression plot of the log dose versus the percentage response was generated, and interpolation (with 95% CIs) was used to determine the laryngeal mask condition. The average of the midpoints of all pairs was used to calculate the ED50 using Dixon’s up-and-down method. The number of responders at each dose was used to plot a sigmoid dose-response curve and a log-dose probit response relation. The other parameters were analysed using repeated measures ANOVA. The patient characteristics are presented as the mean (± SD) or the number (proportion). A *P*-value of < 0.05 was considered to represent significant difference among outcomes. These data were analysed using SPSS version 17.0 (SPSS Inc., Chicago, IL, USA). The sample size of 23 patients was based on α = 0.05 for the two-sided chi-square test to analyse trends in proportions and a logistic model of β = 0.1 to detect the insertion success rates. The sample size of this study also was based on the fact that a minimum of seven independent pairs of participants exhibited a crossover point from a “satisfactory” response to an “unsatisfactory” response. In similar studies in the field of airway device insertion, the number of crossover points varied from six to eight, with six crossover points being the most common. For this study, seven crossovers were sufficient to determine the ED50 of *cis*-atracurium required to insert a laryngeal mask. A minimum of seven pairs of failure-success was required for the statistical analysis. The total score for the insertion conditions was calculated by addition. A score of ≥16 represented the optimal condition for LMA insertion.

## Results

### Patient profile, Haemodynamics, TOF value, and ventilation condition for each dosage

**Fig. 2:**
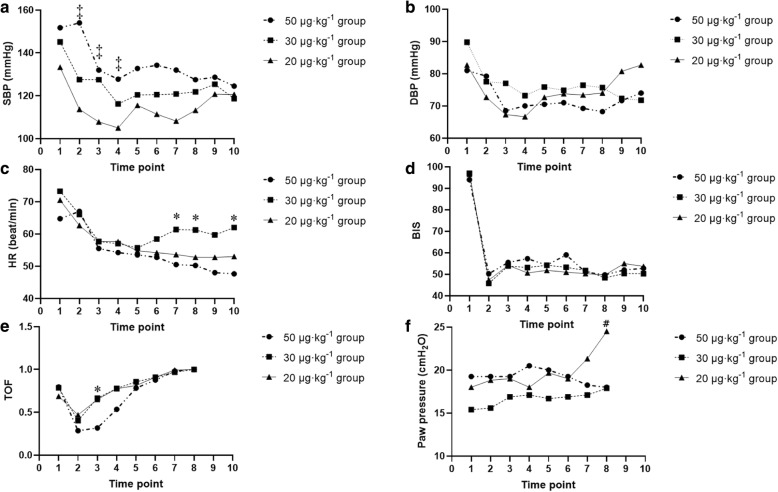
Haemodynamic value fluctuation, train-of-four, Paw pressure and bispectral index value changes in three groups. Data are presented as mean ± SD. * statistical significance between the 50 μg·kg^− 1^ group vs. 30 μg·kg^−1^ group, *P* < 0.05; ‡statistically significant difference between the 50 μg·kg^− 1^ and 20 μg·kg^− 1^ groups, *P* < 0.05; **#** statistically significant difference between the 30 μg·kg^− 1^ and 20 μg·kg^− 1^ groups, *P* < 0.05. There were ten timepoints: T1: administration of *cis*-atracurium, T2: 3 min after administration of *cis*-atracurium (LMA insertion), T3: 10 min after administration of *cis*-atracurium (i.e., beginning of the operation), T4: 20 min after administration of *cis*-atracurium, T5: 30 min after administration of *cis*-atracurium, T6: 40 min after administration of *cis*-atracurium, T7: 50 min after administration of *cis*-atracurium, T8: 60 min after administration of *cis*-atracurium, T9: 70 min after administration of *cis*-atracurium, and T10: 80 min after administration of *cis*-atracurium. Diastolic blood pressure (DBP) did not significantly differ among the groups. Systolic blood pressure (SBP) was significantly decreased in the 50 μg·kg^− 1^ group compared to that in the 20 μg·kg^− 1^ group at T2, T3, and T4 (*P* = 0.0159, *P* = 0.0233, and *P* = 0.0428, respectively). The heart rate (HR) significantly differed among the groups. The HR was significantly decreased in the 50 μg·kg^− 1^ group compared to that in the 20 μg·kg^− 1^ group at T7, T8, and T10 (*P* = 0.0482, *P* = 0.0460, and *P* = 0.0236, respectively). The train-of-four [TOF] onset, the time to TOF ratio = 0 TOF value). The TOF value at T3 was significantly lower in the 50 μg·kg^− 1^ group than in the 30 μg·kg^− 1^ group (*P* = 0.0326). The Paw pressure at each timepoint did not significantly differ between the 50 μg·kg^− 1^ and 30 μg·kg^− 1^ groups. However, the Paw pressure was significantly higher at T8 in the 30 μg·kg^− 1^ group than in the 20 μg·kg^− 1^ group (*P* = 0.0423)

Age, sex, weight, height, BMI, and the ASA Physical Status were similar among the three groups (Table 1). No significant differences were noted in the pH values and nasopharyngeal temperatures in all patients. Haemoglobin values, globulin fraction of plasma protein, and albumin fraction of plasma protein were similar among the groups (Table 1). The preoperative Mallampati variable did not differ among the groups. The operation duration, anaesthesia duration, and blood loss were non-significantly different among groups (Table 1). There was no statistical difference in the DBP among groups. The SBP was significantly decreased in the 20 μg·kg^− 1^ group compared with the 50 μg·kg^− 1^ group at T2, T3, and T4 (*P* = 0.0159, *P* = 0.0233, and *P* = 0.0428, respectively; Fig. 2a and b). For each group, the HR did not significantly differ at most timepoints; however, the HR was significantly decreased at T7, T8, and T10 in the 50 μg·kg^− 1^ group compared to the 30 μg·kg^− 1^ group (*P* = 0.0482, *P* = 0.0460, and *P* = 0.0236, respectively). This finding indirectly reflected the lower stress at these timepoints (Fig. 2c).

**Table 1:** Population Demographics and Intraoperative and Preoperative Data

Variable	50 μg·kg^**− 1**^	30 μg·kg^**− 1**^	20 μg·kg^**− 1**^
Female sex	1 (25)	1 (10)	2 (33.3)
Age (years)	54.50 (14.08)	50.60 (12.65)	48.83 (13.23)
Weight (kg)	77.00 (12.36)	76.90 (5.28)	75.67 (14.90)
Height (cm)	168.75 (7.14)	171.40 (4.97)	169.00 (6.20)
BMI	26.90 (2.37)	26.21 (2.02)	26.32 (3.93)
Haemoglobin (g·L^−1^)	137.5 (19.16)	140.30 (9.59)	138.67 (25.45)
Globin fraction of plasma protein (g·L^−1^)	30.98 (4.89)	24.21 (3.45)	23.87 (5.61)
Albumin fraction of plasma protein (g·L^− 1^)	40.19 (4.24)	39.82 (3.67)	41.22 (6.23)
pH value	7.37 (0.04)	7.38 (0.03)	7.39 (0.03)
Nasopharyngeal temperature (°C)	36.33 (0.25)	36.34 (0.28)	36.38 (0.10)
Operation duration (min)	93 (42.42)	67.20 (41.58)	56.17 (90.27)
Anaesthesia duration (min)	122.25 (41.16)	104.20 (45.85)	99.33 (81.02)
Blood loss (ml)	141.03 (13.52)	139.01 (10.18)	138.89 (20.11)
Mallampati class			
I	0 (0)	1 (10)	1 (16.67)
II	0 (0)	2 (20)	1 (16.67)
III	4 (100)	7 (70)	3 (50)
IV	0 (0)	0 (0)	1 (16.67)
ASA class			
II	2 (50)	2 (20)	1 (16.67)
III	2 (50)	8 (80)	5 (83.33)

*ASA* American Society of Anesthesiologists, *BMI* Body Mass Index

The numerical values (e.g., Mallampati class, ASA score, sex) are expressed as the number (%) or number (proportion). All other values are expressed as the mean (SD)

*P < 0.05; **P < 0.01

The BIS value did not significantly differ among the three groups and was maintained at 40–60 during surgery (Fig. 2d). TOF and airway peak pressure (Paw peak pressure) were recorded at T1-T8, because after T8, muscle relaxation subsided in most cases in the 20 μg·kg^− 1^ group, and participants were unable to tolerate the laryngeal mask. Additional doses of *cis*-atracurium were re-administered to patients after the T8 timepoint. TOF and airway peak pressure (Paw peak pressure) were affected by re-administration of *cis*-atracurium; therefore, we did not include the TOF and airway peak pressure (Paw peak pressure) at T9 and T10 in the statistical analysis. The TOF value at T3 was significantly lower in the 50 μg·kg^− 1^ group than that in the 30 μg·kg^− 1^ group (*P* = 0.0226) (Fig. 2e). The TOF values in the 30 μg·kg^− 1^ group and in the 20 μg·kg^− 1^ group showed no difference at each timepoint, and the TOF values in the 50 μg·kg^− 1^ group and in the 20 μg·kg^− 1^ group also showed no difference at each timepoint (Fig. 2e). The Paw pressure is indicative of airway protection and the supraglottic airway device placement condition. In this study, the airway peak pressure (i.e., mean Paw pressure) did not significantly differ between the 50 μg·kg^− 1^ group and the 30 μg·kg^− 1^ group at any timepoint. However, it was significantly higher at T8 in the 20 μg·kg^− 1^ group than that in the 30 μg·kg^− 1^ group (*P* = 0.0423; Fig. 2f). There were no severe intraoperative haemodynamic changes or adverse reactions such as respiratory depression, nausea, or vomiting in any group.

### The ED50 and ED95 of cis-Atracurium

**Fig. 3:**
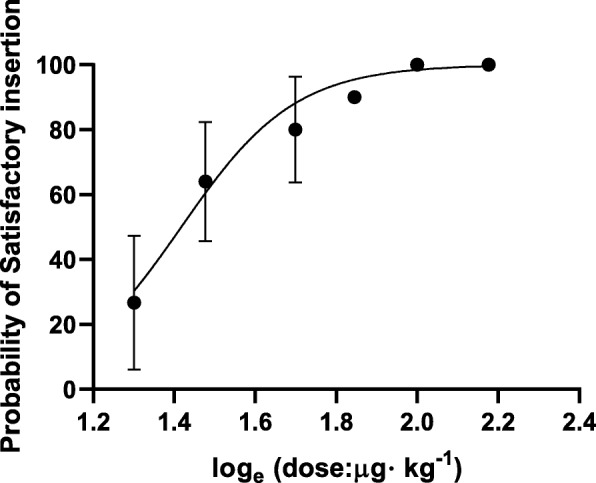
Dose-response curve of *cis*-atracurium for the insertion of the laryngeal mask airway (probability unit vs. concentration). The median effective dose was 26.5 (95% CI: 33.6–29.8). Log dose/probit response curves of *cis*-atracurium for laryngeal mask airway insertion. The points along the lines represent the mean responses of subgroups of ten patients. Only four of six points are provided for the *cis*-atracurium group. In addition, 100% of the patients who received the higher dose (50 μg·kg ^− 1^) had a satisfactory laryngeal mask airway insertion response

In our patients, the ED50 (95% CI) of *cis*-atracurium for laryngeal mask insertion, which was obtained using the up-and-down method, was 26.5 μg·kg^− 1^ (95% CI, 23.6–29.8). We found that the laryngeal mask, based on a probit regression analysis, can be successfully inserted in 50% (95% CI) of anaesthetised adults at a *cis*-atracurium concentration of 26.5 μg·kg^− 1^ (95% CI, 23.6 μg·kg^− 1^-29.8 μg·kg^− 1^). The log dosage probit response curves for *cis*-atracurium for the insertion of the laryngeal mask (probability unit vs. concentration) are shown in Fig. 3. We generated the dose response curves and determined the effective doses of *cis*-atracurium required for the insertion of the laryngeal mask in adult patients (Fig. 3).

## Discussion

In this study, we aimed to determine the optimal cis-atracurium dosage for LMA insertion in anaesthetised adults using Dixon’s up-and-down method and to determine the dose-response curves for LMA insertion in patients undergoing urinary surgery. We found that the ED50 of *cis*-atracurium for LMA insertion in the patients in this study was 26.5 μg·kg^− 1^. Based on our findings, we concluded that laryngeal mask insertion requires the same *cis*-atracurium dosage as tracheal intubation and is an important adjuvant to general anaesthesia. A previous study reported that the calculated ED50 value was 0.0262 mg·kg^− 1^ (95% CI: 0.0258–0.0265) [22]. In another study, the ED50 (SD) and ED95 (SD) values of *cis*-atracurium were 0.021 (0.04) and 0.051 (0.013) mg·kg^− 1^, respectively, for tracheal intubation under total intravenous anaesthesia [23]. Tracheal intubation is required when the neuromuscular response is abolished, which is also an indication for laryngeal mask insertion. No consensus exists regarding the appropriate dose of muscle relaxant required for placing a laryngeal mask; the dose ranges from 1/10 dose to the full dose for normal tracheal intubation [18]. One study [18] also demonstrated that the dose of muscle relaxants required for laryngeal mask placement was smaller than that of the muscle relaxants required for endotracheal intubation. This finding coincided with our results. The estimated ED50 for the total patient group was 35.11 μg·kg^− 1^ [24]. The effective dose in our study differed from the 48 μg·kg^− 1^ that was estimated as the ED95 of *cis*-atracurium by Belmont et al. [25] and differed from the ED50 and ED95 of 30 μg·kg^− 1^ and 53 μg·kg^− 1^, respectively, reported by Lepage et al. [26].

In our study, the SBP was significantly lower in the 20 μg·kg^− 1^ group at T2 (i.e., anaesthesia induction timepoint), T3, and T4, and was indistinguishable from the SBP in the 30 μg·kg^− 1^ group and the 50 μg·kg^− 1^ group. No significant difference in SBP was observed between the 50 μg·kg^− 1^ group and the 30 μg·kg^− 1^ group. However, the propensity to maintain the SBP at a relatively stable level at the preoperative stage was the same among the three groups.

Over 50% of the patients in the 20 μg·kg^− 1^ group had an unsatisfactory first response to laryngeal mask insertion. Placement of a laryngeal mask requires adequate anaesthesia depth and mouth opening. Therefore, administration of additional doses of propofol and sufentanil was needed to achieve satisfactory insertion. The combination of an appropriate dose of muscle relaxants can improve laryngeal mask placement without increasing the incidence of associated adverse reactions, while reducing the amount of propofol or sufentanil anaesthetics and reducing their inhibitory effect on the circulation [27]. Without the use of muscle relaxants, it is necessary to increase the depth of anaesthesia, which prolongs the time of the patient’s recovery of consciousness.

Our results indicated that the HR was significantly decreased in the 50 μg·kg^− 1^ group compared to the 30 μg·kg^− 1^ group at T7, T8, and T10. This finding indirectly reflected a lower stress in the 50 μg·kg^− 1^ group compared to the 30 μg·kg^− 1^ group and the 20 μg·kg^− 1^ group. *Cis*-atracurium did not exert significant haemodynamic changes, even at different concentrations. However, the interaction among anaesthetic agents caused a statistically significant decline in some haemodynamic parameters at certain timepoints. However, this change was not a clinical effect and required no vasopressor agents. Using an appropriate NMBA did not affect the extubation time but reduced the stress reaction [28]. *Cis*-atracurium did not cause harmful autonomic nervous system effects and resulted in reduced secretion of histamine. Some investigators have demonstrated no cardiovascular system variations, even with histamine secretion, when administering a double dose of ED95 to patients with coronary artery disease [29, 30]. The pharmacodynamics of NMBAs are affected by several factors such as temperature [31, 32], use of inhalation agents, magnesium, local anaesthetics [33], antiepileptic drugs, age [34], weight [35], and plasma clearance and volume of distribution [30, 36]. Our study revealed no difference among the three groups in terms of the ASA Physical Status, age, weight, height, and sex. The patients’ body temperature in each group was maintained within the normal range. We restricted the use of inhalation anaesthesia throughout the operation.

Based on our findings, all three groups had recovered to the 100% TOF value by T6. The TOF value was significantly lower in the 50 μg·kg^− 1^ group than in the 30 μg·kg^− 1^ group. This finding indicates that the 50 μg·kg^− 1^ group had more efficient muscle relaxation. At the earliest timepoint, the TOF value did not significantly differ between the 30 μg·kg^− 1^ group and the 20 μg·kg^− 1^ group. The use of a relatively high dose of muscle relaxants will also prolong the extraction of the laryngeal mask due to the prolonged TOF recovery time. The administration of 200% of the ED95 values of *cis*-atracurium, producing an onset duration of 5.2 min, and the time to 25% of T1 recovery at 45 min have been reported [25, 36]. Tulgar [37] demonstrated that the use of subclinical doses of muscle relaxants does not affect the anaesthetic recovery time. In this study, the airway peak pressure (mean Paw pressure) was insignificantly different between the 50 μg·kg^− 1^ group and the 30 μg·kg^− 1^ group at each timepoint. However, the airway peak pressure at T8 was significantly higher in the 20 μg·kg^− 1^ group than in the 30 μg·kg^− 1^ group (*P* = 0.0423). Hemmerling [38] reported that a certain degree of muscle relaxation could prevent reduced sealing of the laryngeal mask owing to recovery of strength in the throat muscles.

Our study was limited in that we did not analyse subgroups of these patients. We plan to determine the differences in the ED50 between younger and elderly patients in our future study because reactions and pharmacokinetics differ between young and elderly patients. We also plan to analyse the differences based on sex in the future. Our research provides information for anaesthesiologists, which could help them improve general anaesthesia induction and LMA insertion in patients undergoing minor surgeries.

## Conclusion

In this study, the ED50 of *cis*-atracurium for effective muscle relaxation in urinary surgery for LMA insertion was 26.5 μg·kg^− 1^.

### Supplementary information

**Additional file 1: Table S1.** Findings Regarding Resistance to Mouth Opening, Resistance to Insertion, Cough, Swallowing, Laryngospasm/Airway Obstruction, and Head and Body Movement.

## Data Availability

The raw data of this study are available from the corresponding author on reasonable request.

## Appendix

### The formula for ED50

The numbers of satisfactory (r) and unsatisfactory cases (s) of laryngeal mask insertion under different doses of *cis*-atracurium were determined. The logarithm (x) of each dose, total number of patients (n), satisfaction rate of laryngeal mask insertion (p), and difference (I) between the logarithms of two adjacent doses were calculated. The ED50 and its 95% CI were calculated using the following formula for half-dose sequential calculation:

$$ \mathit{\mathsf{P}}=\mathsf{r}/\left(\mathsf{r}+\mathsf{s}\right) $$

Pair value lgED50 of ED50 = σ NX/σ n. Standard error of lgED 50 (SlgED50) = I √ σ [p (1–p)/(n–1)] with 95% CI of ED50 pairs of values (lED 50–1.96 SlgED50; lgED50 + 1.96 SlgED50.

## Abbreviations

ASA: American Society of Anesthesiologists
BIS: Bispectral index
BMI: Body Mass Index
DBP: Diastolic blood pressure
ED50: The median effective dose
HR: Heart rate
IRB: Institutional review board
LMA: Laryngeal mask airway;
NMBAs: Neuromuscular blocking agents
SBP: The systolic blood pressure
SpO_2_: Pulse oxygen saturation
STROBE: Observational Studies in Epidemiology
TOF: Train-of-four
