# Supplementary material for: The median effective dose (ED50) of cis-Atracurium for laryngeal mask airway insertion during general Anaesthesia for patients undergoing urinary surgery
Source: BMC Anesthesiol. 2020 Mar 19;20:68. doi: 10.1186/s12871-020-00982-3 (PMC7081559; doi:10.1186/s12871-020-00982-3)
Supplement: Supplementary file 1 — Additional file 1: Table S1. Findings Regarding Resistance to Mouth Opening, Resistance to Insertion, Cough, Swallowing, Laryngospasm/Airway Obstruction, and Head and Body Movement. [file 12871_2020_982_MOESM1_ESM.docx]

**Table S1**

Findings Regarding Resistance to Mouth Opening, Resistance to Insertion, Cough, Swallowing, Laryngospasm/Airway Obstruction, and Head and Body Movement

| Objection | Score | Standard |
| --- | --- | --- |
| Resistance to mouth opening grade | 3 | No |
|  | 2 | Significant |
|  | 1 | Undue force required |
| Resistance to insertion grade | 3 | Easy |
|  | 2 | No |
|  | 1 | Significant |
| Cough | 3 | Undue force required |
|  | 2 | Light |
|  | 1 | Serious |
| Swallowing grade | 3 | None |
|  | 2 | Slight |
|  | 1 | Gross |
| Laryngospasm/airway obstruction | 3 | None |
|  | 2 | Slight |
|  | 1 | Gross |
| Head and body movement | 3 | None |
|  | 2 | Slight |
|  | 1 | Gross |

The evaluation criteria of laryngeal mask placement conditions comprise six-three levels proposed by Sivalingam, which are graded on six aspects: resistance to mouth opening, resistance to insertion, coughing, swallowing, laryngospasm/airway obstruction, and head and body movement. Each item is divided into 3 points, 2 points, and 1 point, based on the degree of severity. The full score was 18 points; 16 points and higher indicated a satisfactory response, and less than 16 points indicated an unsatisfactory response.
